# Barriers to Seeking Medical Care for Hemorrhoidal Symptoms: A Cross-Sectional Observational Study

**DOI:** 10.3390/jcm14155361

**Published:** 2025-07-29

**Authors:** Adrian Cote, Roxana Loriana Negrut, Bogdan Feder, Ioan Andrei Antal, Maur Sebastian Horgos, Emilia Tomescu, Adrian Marius Maghiar

**Affiliations:** 1 Department of Surgical Disciplines, Faculty of Medicine and Pharmacy, University of Oradea, 410073 Oradea, Romania; adrian.cote@didactic.uoradea.ro (A.C.); bogdanfeder@yahoo.com (B.F.); ioan.antal@yahoo.com (I.A.A.); mauhorgos@yahoo.com (M.S.H.); amaghiar@uoradea.ro (A.M.M.); 2 County Clinical Emergency Hospital Bihor, 410087 Oradea, Romania; emilia_tomescu96@yahoo.com

**Keywords:** hemorrhoids, symptom perception, health-seeking behavior, public awareness, stigma

## Abstract

**Background:** Despite their high prevalence and potential for significant morbidity, hemorrhoidal symptoms remain underreported and undertreated. Misconceptions and stigma may delay care-seeking behaviors and negatively influence patient outcomes. **Methods:** We conducted a cross-sectional, questionnaire-based study in Romania to assess public awareness, attitudes, and barriers related to hemorrhoidal disease. The survey included 185 participants and evaluated variables such as symptom severity, understanding of the condition, perceived stigma, and willingness to consult a physician. **Results:** Only 30.8% of participants had sought medical advice for hemorrhoidal symptoms. Younger age (*p* < 0.001), male sex (*p* = 0.013), and lower levels of perceived severity were significantly associated with reluctance to seek medical care. The most frequently reported barriers were embarrassment and fear of invasive diagnostic procedures. Colonoscopy and digital rectal examination were identified as major deterrents by 39.5% and 38.9% of respondents, respectively. Educational level influenced both the perceived understanding of the disease (*p* = 0.001) and comfort in discussing anal symptoms (*p* = 0.002). Gender preference for physicians was significantly associated with respondent sex (*p* = 0.007) but not with education or age. **Conclusions:** Hemorrhoidal disease remains a stigmatized and underestimated condition. Public health efforts should prioritize educational interventions, destigmatization campaigns, and improved physician–patient communication to facilitate earlier diagnosis and better disease management.

## 1. Introduction

Hemorrhoidal disease is a highly prevalent anorectal condition that affects millions of people worldwide and represents a major medical and socioeconomic problem; however, its true prevalence in the general population remains unclear, partly because many patients do not seek medical treatment despite the condition being one of the most encountered anorectal disorders in general practice [1,2,3,4]. Despite its benign nature and the availability of effective conservative and surgical treatments, many individuals avoid seeking medical advice when experiencing anorectal symptoms. This delay in presentation may result in unnecessary suffering, progression of disease, and increased healthcare burden.

Multiple factors contribute to this reluctance, including embarrassment, fear of diagnosis or treatment, lack of time, and limited awareness about the condition. Cultural and social norms, particularly regarding anal health, further exacerbate the stigma, especially in conservative societies. Additionally, gender dynamics between patients and physicians may influence the willingness to discuss such intimate symptoms, often resulting in deferred or avoided medical consultations.

While previous studies have explored health-seeking behaviors in gastrointestinal and colorectal conditions, data specifically focused on hemorrhoidal symptoms remain scarce—particularly in Eastern European populations. Understanding the psychosocial and practical barriers that prevent patients from consulting a physician is essential for developing targeted public health strategies, improving patient education, and facilitating timely access to care.

Although a recent international online survey conducted in eight countries, including Romania, estimated a hemorrhoidal disease prevalence of approximately 11%, the data collected were primarily limited to symptom frequency and treatment types, without exploring patients’ perceptions or help-seeking behaviors [5]. To date, no Romania-specific study has investigated the psychosocial or practical barriers that prevent individuals from consulting a physician for anorectal symptoms.

This study aims to fill this gap by identifying and quantifying the perceived barriers to seeking medical care for hemorrhoidal symptoms in a Romanian population. Using a structured, anonymous online questionnaire, we evaluated symptom burden, attitudes, knowledge, and stigma-related factors across a demographically diverse adult cohort. The findings aim to inform both public health education and clinical communication strategies for improving early presentation and care in benign anorectal disease.

The present study aims to identify and analyze the most common self-reported barriers that hinder individuals from seeking medical consultation for hemorrhoidal symptoms. Through a structured online questionnaire distributed among Romanian adults, we evaluated the relationship between symptom burden, perceived stigma, and health-seeking behavior in this context.

## 2. Materials and Methods

### 2.1. Study Design and Population

This cross-sectional observational study was conducted using an anonymous, self-administered online questionnaire designed to evaluate the perceived barriers to seeking medical consultation for hemorrhoidal symptoms. The survey targeted adult participants aged 18 years and above who could understand the Romanian language. Data were collected between 13 April and 16 June 2025. The objective was to assess the perceived barriers that prevent individuals from seeking medical care for hemorrhoidal symptoms, with a focus on psychosocial, cultural, and systemic factors.

### 2.2. Questionnaire Development

The questionnaire was created using Google Forms (Google LLC, Mountain View, CA, USA) and consisted of 23 items covering the following domains:

Sociodemographic data: age, sex, place of residence, education level, and childbirth history (for females).

Experience with hemorrhoidal symptoms: type, frequency, severity, and perceived impact.

Healthcare behavior: whether medical consultation was sought and reasons for avoidance.

Perceptions and beliefs: knowledge of hemorrhoids, perceived seriousness, embarrassment, physician gender preference.

Barriers: multiple-choice items assessing factors such as fear, shame, time constraints, and financial reasons.

The questionnaire required approximately 5–10 min to complete and was disseminated voluntarily through social media and institutional networks.

The questionnaire was pre-tested on a small group of individuals (*n* = 60) to ensure clarity, structure, and language appropriateness. Minor revisions were made accordingly.

### 2.3. Ethical Considerations

Participation was entirely voluntary, anonymous, and without compensation. An informed consent statement was included at the beginning of the questionnaire, explicitly stating that the data would be used strictly for scientific purposes and that no identifiable information would be collected. Formal Ethical Subcommittee approval was received from the University of Oradea (No. 19/29.05.2025). One of the principal investigators (Roxana Loriana Negruț) successfully completed the certified training course Protecting Human Research Participants provided by PHRP Online Training, Inc. (National Institutes of Health (NIH), Bethesda, MD, USA; Certification No. 3004618, completed on 13 April 2025), ensuring compliance with good clinical and research practice standards.

### 2.4. Statistical Analysis

All responses were exported to Microsoft Excel (Version 16.98; Microsoft Corp., Redmond, WA, USA) and analyzed using JASP software (version 0.19.3 Apple Silicon, JASP Team, Amsterdam, The Netherlands), and statistical significance was set at a *p*-value of <0.05. Descriptive statistics were used to characterize the study population, including frequencies and percentages for categorical variables and medians with interquartile ranges for ordinal and non-normally distributed continuous variables.

To examine associations between the presence of medical consultation and sociodemographic or clinical variables, the following statistical methods were employed:

Chi-square tests were used to assess associations between categorical variables such as sex, education level, and individual hemorrhoidal symptoms (e.g., pain, bleeding, and swelling) in relation to medical consultation.

Mann–Whitney U tests were conducted to compare ordinal variables (e.g., frequency of symptoms, perceived severity, and symptom burden) between those who sought medical care and those who did not. Effect sizes were reported using rank biserial correlations, with 95% confidence intervals.

Spearman’s rank correlation coefficients (ρ) were used to evaluate the relationships between ordinal variables, such as perceived understanding of the disease, perception of severity, social openness to discussion, and likelihood of recommending medical consultation.

Kruskal–Wallis tests were applied to compare ordinal variables (e.g., perceived comfort in discussing symptoms and understanding of the condition) across multiple education levels.

Binary logistic regression was used to identify independent predictors of medical consultation, with age, sex, education level, and perception of disease severity entered as covariates. Model fit was assessed using the Akaike Information Criterion (AIC), Nagelkerke R^2^, and the Wald test for individual predictors.

Data preprocessing included recoding categorical responses into ordinal or binary formats appropriate for the tests used. No imputation was performed for missing data; only complete responses were analyzed.

Generative artificial intelligence (ChatGPT, OpenAI version 4.0) was used to assist in the refinement of the English used, abstract formulation, and phrasing improvements throughout the manuscript. No content, data, or results were generated by AI. All scientific content, study design, analysis, and interpretation were conducted by the authors.

## 3. Results

### 3.1. Participant Characteristics

A total of 185 individuals completed the questionnaire. The mean age of respondents was 41.5 years (range: 19–91 years). Of them, 127 (68.6%) were female and 58 (31.4%) were male. Most participants resided in urban areas (82.2%), while 17.8% reported living in rural settings.

Regarding their educational background, 55.1% had completed university studies, and an additional 29.7% held postgraduate degrees. A smaller proportion had only secondary education (10.3%), primary education (4.3%), or post-secondary non-university education (0.5%). The characteristics of study participants are detailed in Table 1.

Out of 185 respondents, 65.9% reported having experienced symptoms that they associated with hemorrhoidal disease, while 25.4% stated that they had not, and 8.6% were uncertain. Despite the high rate of self-reported symptoms, only 30.8% of those affected sought medical consultation. The remaining 69.2% did not consult a healthcare professional, indicating a substantial gap between symptom recognition and medical engagement.

**Figure 1:**
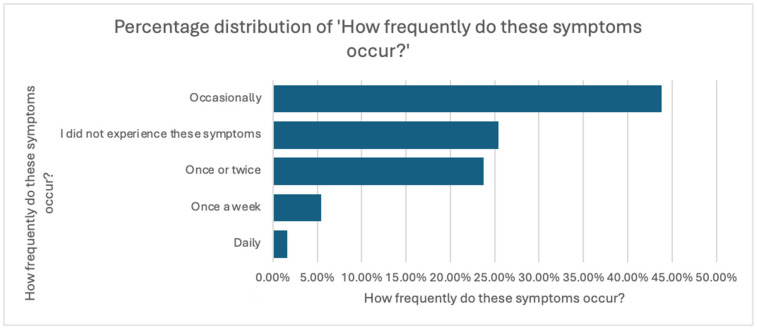
Distribution of Symptom Frequency in the Study Population.

**Figure 2:**
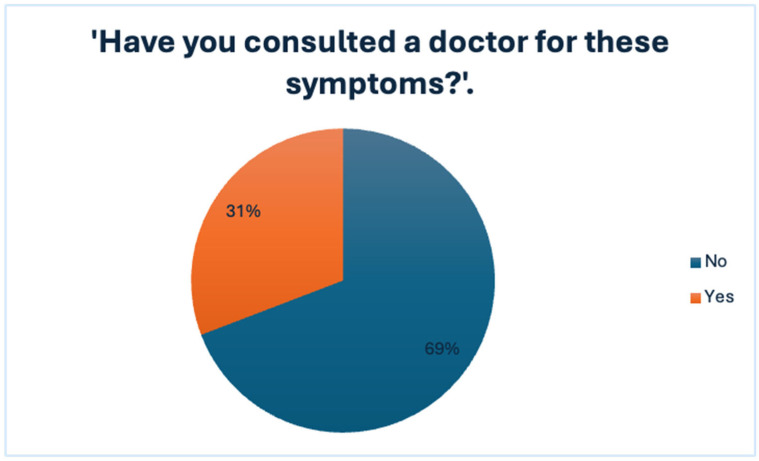
Medical Consultation Rates Among Individuals Reporting Symptoms.

Notably, this avoidance occurred even though the symptoms were present and recurrent, as further explored in the subsequent analysis of symptom frequency and perceived impact on daily life, presented in Figure 1 and Figure 2. These findings suggest that symptom burden alone may not be sufficient to prompt medical consultation and point toward additional psychological or social barriers.

### 3.2. Symptom Characteristics

Among respondents who reported experiencing hemorrhoidal symptoms, the most cited were the following:

- Anal swelling (44.3%)
- Anal pain during defecation (40.5%)
- Anal itching (37.8%)
- Rectal bleeding (30.3%)

Only a small proportion (21.1%) selected “None of the above,” indicating most respondents could identify at least one typical symptom.

Regarding frequency, 43.8% of symptomatic participants reported symptoms occurring occasionally, 23.8% experienced them only once or twice, while 5.4% and 1.6% experienced symptoms weekly or daily, respectively. Interestingly, 25.4% indicated that they had never experienced such symptoms.

**Figure 3:**
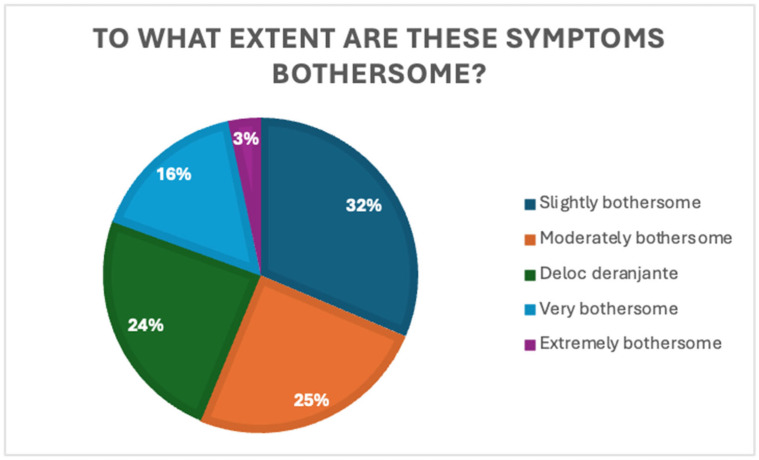
Distribution of self-reported symptom burden.

Despite the low rate of medical consultation, the perceived impact of symptoms was notable: 31.4% of respondents found the symptoms mildly bothersome, 24.9% reported them as moderately bothersome, and 16.2% reported them as very bothersome. Only 3.2% rated their symptoms as extremely disturbing, while only 24.3% stated that the symptoms did not interfere at all with their daily lives. Figure 3 shows the distribution of perceived symptom burden.

These findings indicate that even mild or occasional symptoms exert a non-negligible impact on daily functioning, yet do not necessarily translate into increased healthcare-seeking behavior.

### 3.3. Awareness and Perception of Hemorrhoidal Disease

When asked about the perceived understanding of hemorrhoids, 45.9% rated their understanding as very good (score 5), and 19.5% gave a score of 4. Conversely, 10.8% indicated minimal understanding (score 1), and 5.9% chose score 2.

**Figure 4:**
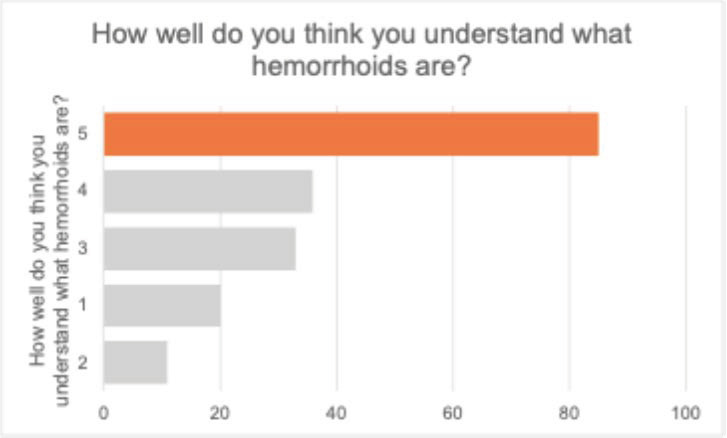
Self-assessed understanding of hemorrhoids among respondents.

These results, presented in Figure 4, suggest that nearly two-thirds of respondents (65.4%) perceive themselves as having good-to-excellent knowledge of the condition. However, a non-negligible proportion still reported limited understanding, which may affect their health behaviors and perceptions.

**Figure 5:**
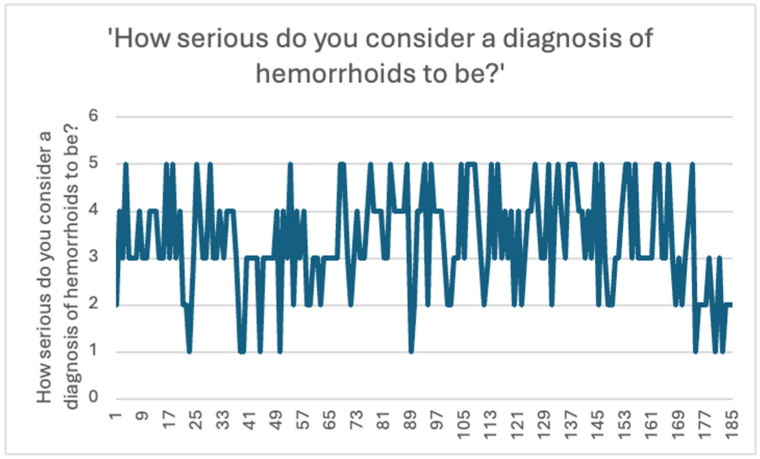
Perceived seriousness of a hemorrhoid diagnosis among respondents.

Regarding participants’ perceived severity of a hemorrhoid diagnosis, participants responded on a 5-point Likert scale (1 = not severe at all, 5 = very severe). Most respondents (38.4%) rated the condition as moderately severe (score 3), while 23.2% selected 4 and 18.4% selected 5. Only 4.9% considered the condition as not severe at all (score 1). The data is presented in Figure 5.

These findings suggest that although hemorrhoidal disease is perceived as a moderate-to-severe condition by most participants, there remains a degree of variability in individual perceptions, which may influence their care-seeking behavior.

**Figure 6:**
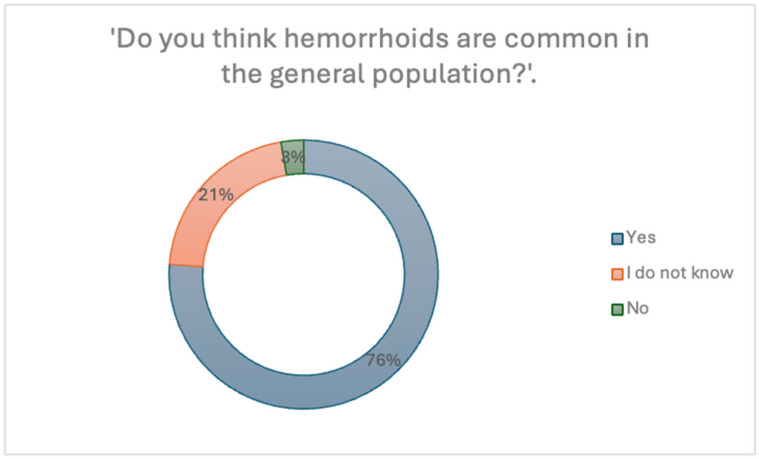
Perception of the prevalence of hemorrhoids in the general population.

When asked whether they believe hemorrhoids are common in the general population, many respondents (76.2%) answered “Yes”, while 21.1% stated “I don’t know”, and only 2.7% answered “No”, as shown in Figure 6. This suggests a relatively high level of public awareness regarding the prevalence of hemorrhoidal disease.

Spearman’s rank-order correlations, presented in Table 2, were used to explore relationships between participants’ self-reported understanding of hemorrhoids and various attitudinal variables:

- A weak but significant positive correlation was found between understanding of hemorrhoids and perceived disease severity (ρ = 0.245, *p* < 0.001).
- A moderate positive correlation was observed between understanding and the likelihood of recommending medical consultation to a close person (ρ = 0.515, *p* < 0.001), indicating that those with greater understanding were more inclined to promote professional care.
- There was a significant negative correlation between understanding and the belief that hemorrhoids are common in the general population (ρ = −0.334, *p* < 0.001), suggesting that better-informed individuals may perceive the condition as less widespread. This indicates that participants who believed they understood the condition well were slightly less likely to perceive it as frequent—a potential misconception worth addressing in patient education.
- No significant association was found between understanding and the use of over-the-counter treatments (ρ = −0.109, *p* = 0.141).

Participants’ perceptions of the severity and understanding of hemorrhoidal disease varied significantly across demographic groups.

Sex differences were assessed using a Mann–Whitney U test. Results, shown in Table 3, indicated a statistically significant difference between men and women in how severe they perceived hemorrhoidal disease to be (U = 4345.500, *p* = 0.041). Women tended to rate the condition as more severe compared to men.

Additionally, a Kruskal–Wallis test was used to examine differences in perceived understanding of the disease across educational levels. Results are presented in Table 4. The test showed a statistically significant difference (H(4) = 18.341, *p* = 0.001), indicating that individuals with higher education levels reported a better understanding of what hemorrhoids are.

Among the 121 respondents who reported understanding hemorrhoids either “well” or “very well,” 81 (66.9%) reported having experienced anorectal symptoms. However, only 40 of them (33.1%) consulted a doctor. Among those who did not seek medical consultation (n = 81), the most reported reason was the belief that symptoms would resolve spontaneously (28.39%). Other reported barriers included lack of time (18.51%), shame or embarrassment (14.81%), fear of a serious diagnosis (6.17%), fear of surgery (4.93%), not finding a physician of the same sex (2.46%), and not knowing where to go (1.23%). Financial reasons were rarely reported (1.23%), and 45.67% selected “other” reasons.

### 3.4. Predictors of Medical Consultation for Hemorrhoidal Symptoms

A binary logistic regression was performed to identify factors associated with the likelihood of having consulted a physician for hemorrhoidal symptoms. The dependent variable was whether the participant reported having seen a physician (1 = Yes, 0 = No). The independent variables included age, sex, perceived disease severity, and education level.

The regression model was statistically significant (χ^2^ = 19.900, *p* = 0.006), suggesting that the included predictors reliably distinguished between those who sought medical advice and those who did not. The model explained approximately 14.4% of the variance (Nagelkerke R^2^ = 0.144). The results are summarized in Table 5 and Table 6.

- Age was significantly associated with medical consultation (OR = 1.037, *p* = 0.010), indicating a slightly increased likelihood of consultation with increasing age.
- Sex was also significant (OR = 2.46, *p* = 0.013), with females being more likely than males to seek medical help.
- Perceived severity of hemorrhoidal disease did not significantly predict consultation behavior (*p* = 0.242).
- Educational level was not a significant predictor in the model (*p* > 0.05 for all categories compared to the reference).

To further investigate potential behavioral predictors, we assessed whether the frequency of symptoms was associated with the decision to seek medical advice. An Independent Samples *T*-Test (Mann–Whitney U test) revealed a significant difference between individuals who consulted a physician and those who did not in terms of how often they experienced symptoms (U = 2452.5, *p* < 0.001) (Table 7).

The rank-biserial correlation was −0.328 (95% CI: −0.478 to −0.158), indicating a moderate effect size. This suggests that individuals experiencing more frequent hemorrhoidal symptoms were significantly more likely to have sought medical care.

### 3.5. Sociodemographic Differences in Medical Consultation

**Figure 7:**
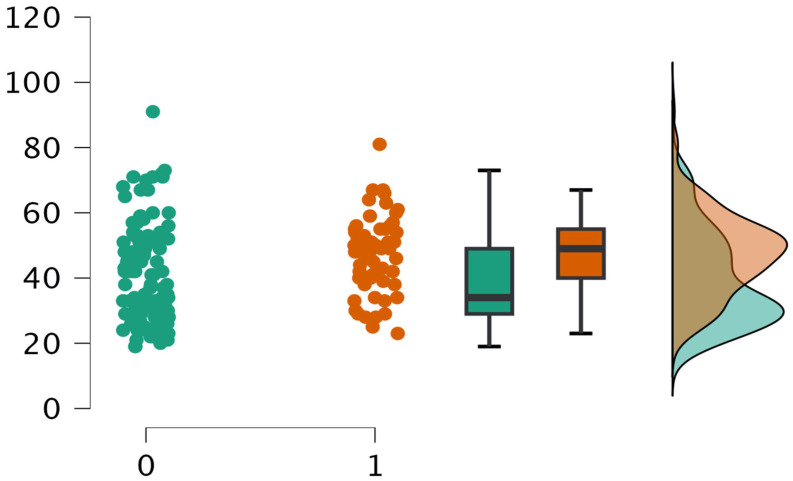
Age distribution by medical consultation status.

Age was a significant factor associated with the likelihood of consulting a physician. An independent samples *t*-test showed that individuals who sought medical attention were significantly older than those who did not (*t*(182) = −3.40, *p* < 0.001). The distribution of ages, illustrated in Figure 7, indicates a tendency for younger respondents to avoid medical consultation, while older participants were more likely to seek professional advice for their symptoms.

This may reflect differences in health-seeking behavior, perceived vulnerability, or symptom interpretation across age groups.

A Chi-square test of independence was conducted to examine the association between gender and medical consultation for hemorrhoidal symptoms. The results revealed a statistically significant relationship between sex and the likelihood of seeking medical advice (χ^2^(1) = 4.427, *p* = 0.035). Women (coded as 1) were more likely to report having consulted a physician compared to men.

A Chi-square test was performed to assess the relationship between participants’ level of education and their decision to seek medical consultation for hemorrhoidal symptoms. The test did not reveal a statistically significant association between educational level and medical consultation (χ^2^(4) = 4.299, *p* = 0.367), suggesting that education was not a predictor for seeking medical advice in this sample.

### 3.6. Symptoms Associated with Seeking Medical Consultation

We performed chi-square tests to identify which hemorrhoid-related symptoms were significantly associated with seeking medical care. The following symptoms showed statistically significant associations with consultation:

- Pain during defecation was significantly associated with medical consultation (χ^2^(1) = 10.29, *p* = 0.001).
- Anal swelling also showed a significant association (χ^2^(1) = 10.39, *p* = 0.001).
- Anal itching was significantly associated with consultation (χ^2^(1) = 7.67, *p* = 0.006).
- Discomfort while sitting was a strong predictor (χ^2^(1) = 11.20, *p* < 0.001).
- Reporting no symptoms at all was inversely associated with seeking care (χ^2^(1) = 17.81, *p* < 0.001).

In contrast, rectal bleeding, mucous discharge, sensation of incomplete evacuation, and reporting other symptoms were not significantly associated with consultation behavior (all *p* > 0.05).

These findings suggest that certain symptoms perceived as more disruptive to daily life (e.g., pain, swelling, and discomfort) are more likely to prompt individuals to seek medical advice, compared to those considered more “benign” or familiar.

A Mann–Whitney U test was conducted to assess whether the perceived impact of symptoms on daily life was associated with seeking medical consultation. The results, presented in Table 8, revealed a statistically significant difference between the two groups (U = 1973.00, *p* < 0.001), with a moderate-to-strong effect size (rank-biserial correlation = −0.459, 95% CI [−0.590, −0.305]).

This indicates that participants who considered their symptoms more bothersome in daily life were significantly more likely to have sought medical advice.

### 3.7. Barriers to Seeking Medical Consultation

**Figure 8:**
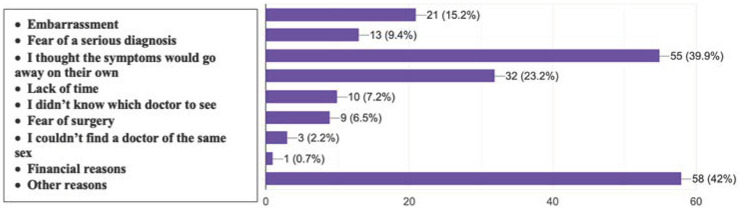
Self-reported reasons for not seeking medical consultation for hemorrhoidal symptoms among survey respondents (multiple answers allowed).

Among respondents who reported not consulting a physician for their hemorrhoidal symptoms (n = 138), the most frequently cited reasons were as follows (Figure 8):

- The belief that symptoms would resolve on their own (39.9%);
- Lack of time (23.2%);
- Feelings of shame (15.2%);
- Fear of a serious diagnosis (9.4%).

Other less frequently reported barriers included not knowing which specialist to consult (7.2%), fear of surgery (6.5%), inability to find a same-sex physician (2.2%), and financial concerns (0.7%).

Notably, 42% of respondents selected “Other reasons”, suggesting the presence of additional, potentially complex psychosocial or contextual barriers.

These findings emphasize the need for improved public education and destigmatization regarding anorectal conditions to encourage timely medical engagement.

**Figure 9:**
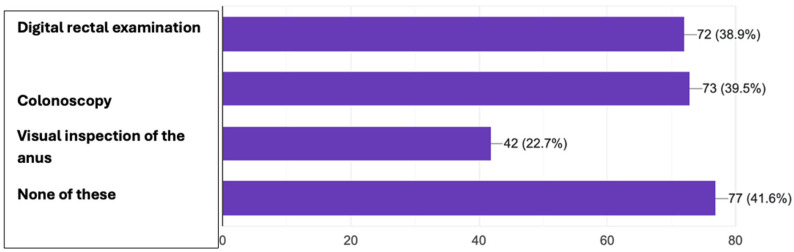
Reported barriers to seeking medical consultation for hemorrhoidal symptoms.

When asked which types of investigations might deter them from seeking medical care, participants most frequently indicated colonoscopy (39.5%) and digital rectal examination (38.9%) as procedures associated with hesitation, as seen in Figure 9. Visual inspection of the anus was a reported deterrent by 22.7% of respondents. Notably, 41.6% of participants selected “None of the above”, indicating that for a considerable portion of the population, diagnostic procedures alone may not constitute a barrier to medical consultation.

These findings suggest that although some anorectal procedures are perceived as invasive or embarrassing, they are not universally seen as obstacles, highlighting the need for better patient education and reassurance regarding the nature and importance of these investigations.

### 3.8. Self-Medication and Barriers to Medical Consultation

When asked about the use of non-prescription treatments for their symptoms, more than half of the respondents (57.8%) reported having used such remedies. The most common source was over-the-counter medications from pharmacies (45.4%), followed by natural remedies (10.8%) and online recommendations (1.6%). Meanwhile, 42.2% of participants reported not using any non-prescription treatment.

These findings suggest a notable reliance on self-medication, particularly via pharmacy products, which may reflect a preference for self-management or a reluctance to seek professional help.

### 3.9. Communication-Related Barriers

Despite the high prevalence of symptoms, not all individuals feel comfortable discussing anorectal issues with a physician. When asked whether they would feel comfortable talking about anal symptoms with a doctor, only 70.8% answered “Yes”, while 11.4% answered “No” and 17.8% were unsure.

This indicates that for nearly 30% of respondents, communication discomfort may act as a significant barrier to medical consultation. Such stigma may delay diagnosis and appropriate treatment.

A Kruskal–Wallis test was conducted to evaluate whether the level of education influences respondents’ comfort in discussing anorectal symptoms with a healthcare provider. The analysis revealed a statistically significant difference across education groups, χ^2^(2) = 12.342, *p* = 0.002. Results are presented in Table 9.

This finding suggests that lower educational attainment may be associated with greater discomfort in discussing these symptoms, underlining the role of health literacy and communication training in public health strategies. In contrast, sex was not found to be a statistically significant factor influencing communication comfort, suggesting that men and women reported similar levels of ease or discomfort when addressing anorectal complaints with healthcare providers.

### 3.10. Patient Preferences Regarding Physician Gender

**Figure 10:**
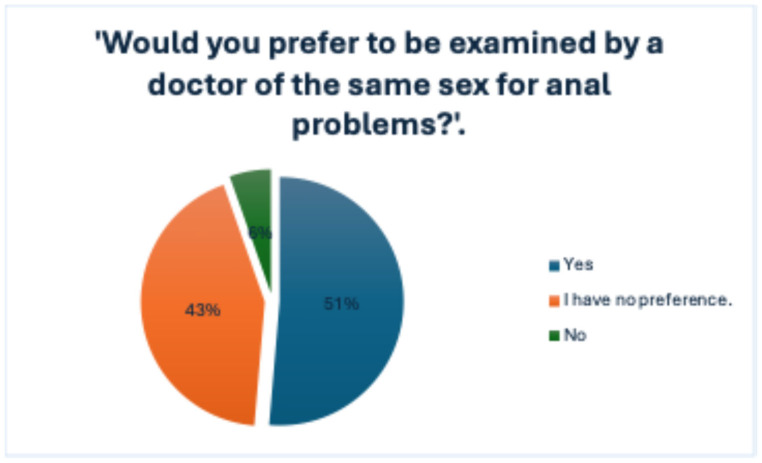
Participant preferences regarding the sex of the physician for consultation about anal symptoms.

When directly asked whether they would prefer to be examined by a physician of the same sex for anorectal issues, 51.4% of respondents answered affirmatively, while 43.2% stated that they had no specific preference, and only 5.4% indicated a clear opposition to such a preference, as shown in Figure 10.

**Figure 11:**
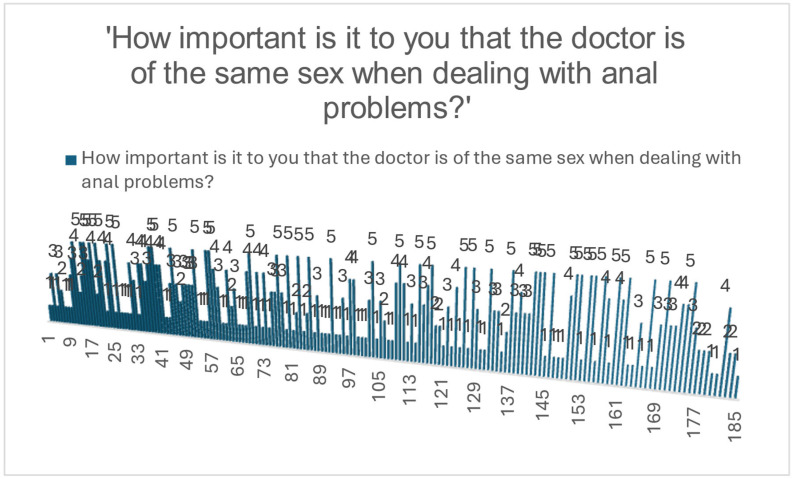
Distribution of perceived importance regarding physician sex when addressing anal problems.

In assessing factors that may influence patient comfort when addressing anorectal symptoms, participants were asked to rate the importance of the physician being of the same sex. Responses varied, with 35.1% of respondents indicating that this factor was not important at all (score 1), while 22.2% rated it as very important (score 5). Overall, 37.3% of participants rated this criterion with a score of 4 or 5, suggesting that for a significant subset of individuals, physician gender may impact their willingness to seek care or communicate openly, as illustrated in Figure 11.

The preference for a same-sex physician was significantly associated with the respondent’s sex (χ^2^ = 7.367, *p* = 0.007). This suggests that while not all participants explicitly rate physician gender as important, sex-matched preference is more likely to be expressed by females, potentially influencing medical-seeking behavior in anorectal conditions.

When analyzed in relation to education level, no statistically significant association was found (χ^2^ = 1.071, df = 4, *p* = 0.899). This indicates that educational attainment did not significantly influence respondents’ preference for a same-sex physician in the context of anorectal health concerns.

No significant association was found between the participants’ age and their preference for being examined by a physician of the same sex (Mann–Whitney U = 513.000, *p* = 0.682).

### 3.11. Willingness to Recommend Medical Evaluation

Most participants indicated a high likelihood of recommending medical evaluation to someone close experiencing hemorrhoidal symptoms, with 60% selecting the highest score (5 on a 5-point Likert scale).

## 4. Discussion

This study provides important insights into the knowledge, perceptions, and health-seeking behaviors of Romanian adults regarding hemorrhoidal disease. Despite the high prevalence of symptoms, a substantial proportion of respondents reported not consulting a physician, underscoring the need to better understand the barriers and predictors of medical consultation.

A key finding was that perceived frequency and impact of symptoms significantly influenced the likelihood of seeking care. Respondents who reported more frequent symptoms (Mann–Whitney U = 2452.5, *p* < 0.001, *r_s_* = −0.328) and those who perceived their symptoms as more disturbing to daily life (Mann–Whitney U = 1973, *p* < 0.001, *r_s_* = −0.459) were significantly more likely to seek medical attention. Specific symptoms such as anal pain (χ^2^ = 10.29, *p* = 0.001), swelling (χ^2^ = 10.39, *p* = 0.001), itching (χ^2^ = 7.67, *p* = 0.006), and sitting discomfort (χ^2^ = 11.2, *p* < 0.001) were also positively associated with medical consultation.

Conversely, the presence of rectal bleeding, often considered a hallmark symptom of hemorrhoidal disease, was not significantly associated with medical presentation (χ^2^ = 0.37, *p* = 0.545). This surprising result may suggest a normalization of this symptom among the population or confusion with other benign conditions. A cross-sectional survey in Makkah (n = 495) found that pain (78%) and discomfort (43%) were the primary motivators for seeking care [6]. A study in pregnant women found that symptoms like anal pain, pruritus, and mucus discharge impact quality of life and motivate healthcare-seeking behavior, but awareness and discussion remain limited [7].

In the current study, feelings of shame were reported in 15.2% of those who did not seek medical care. A cross-sectional survey from the Makkah region in Saudi Arabia found that embarrassment was a major reason for *delayed* presentation, even though many recognized their symptoms as significant [6]. Similarly, an exploratory study among the Bisha population in Saudi Arabia reported that 29.6% felt embarrassed to see a doctor, and cultural/language factors played a significant role in delaying care [8]. Similar dynamics regarding patient reluctance, stigma, and the role of health literacy in accessing care have also been observed in Romanian studies addressing infertility and intimate health conditions [9,10]. A qualitative study from Brazil highlights that embarrassment causes patients to delay seeking care, describing how hemorrhoids are perceived as “unhygienic and unclean” and often lead to postponement of hospital visits [11].

An international online survey, including respondents from Romania, reported a hemorrhoid prevalence of ~11%, with most cases being mild. Those who consulted a physician were more likely to undergo interventions and medication [5]. Our study complements these findings by analyzing why many do or do not seek that care, focusing on barriers and predictors.

Younger age was associated with a lower likelihood of consulting a physician (Student’s *t* = −3.400, *p* < 0.001), and logistic regression confirmed that age (*p* = 0.010) and sex (*p* = 0.013) were significant predictors of medical consultation. However, the perception of disease severity (*p* = 0.242) and education level did not reach significance in the regression model. Although female sex was associated with higher consultation rates (χ^2^ = 4.43, *p* = 0.035), this factor lost significance in multivariate analysis. A study of hospital records in Afghanistan suggested significant male predominance in hemorrhoid cases, hinting at possible social or cultural factors influencing who seeks treatment [12]. Our finding that women are more likely to seek consultation and prefer same-sex physicians for sensitive exams resonates with this culturally influenced gender behavior.

Regarding knowledge and awareness, there was a weak but statistically significant positive correlation between perceived understanding of hemorrhoids and the perceived seriousness of the condition (*r_s_* = 0.245, *p* < 0.001), and a stronger correlation with the likelihood of recommending a medical visit to others (*r_s_* = 0.515, *p* < 0.001). Conversely, a negative correlation was found between perceived understanding and the belief that hemorrhoids are common (*r_s_* = −0.334, *p* < 0.001), suggesting that increased knowledge may be associated with a more nuanced understanding of disease impact. The finding that 66.9% of informed respondents reported symptoms—but only one-third of them sought medical care—underscores the disconnect between knowledge and behavior. The most common reason for non-presentation was the belief that symptoms were self-limiting. This reflects a tendency to trivialize anorectal symptoms even among those who are medically informed. Psychosocial and behavioral barriers such as shame, time constraints, or fear also played important roles and should be addressed in public awareness efforts.

Notably, educational level was not associated with health-seeking behavior (χ^2^ = 4.30, *p* = 0.367), but it was significantly related to perceived understanding of hemorrhoids (Kruskal–Wallis *H* = 18.34, *p* = 0.001) and how comfortable individuals felt discussing anal symptoms with a physician (Kruskal–Wallis *H* = 12.34, *p* = 0.002). These findings suggest that communication barriers may persist even in more educated populations and highlight the importance of clinician-initiated dialogue.

Sex-related preferences also emerged. A significantly higher proportion of women preferred being examined by a physician of the same sex (χ^2^ = 7.37, *p* = 0.007), a factor which should be considered in the organization of proctologic services. However, this preference was not significantly associated with either age (*p* = 0.682) or education level (χ^2^ = 1.07, *p* = 0.899), suggesting that gender concordance may have intrinsic importance for some patients, independent of sociodemographic variables.

A recent study published in 2023 demonstrated that quality of life decreases significantly with increasing symptom burden in hemorrhoidal patients, reinforcing the clinical relevance of symptoms such as pain, discomfort, and hygiene concerns [13].

Together, these results emphasize that while knowledge and symptom severity influence consultation behavior, social, psychological, and gender-related factors also play a central role. Case-based research has previously emphasized the complexity of managing sensitive medical conditions during pregnancy and the need for nuanced patient-centered communication [14]. Interventions aiming to reduce stigma, normalize discussion around anorectal symptoms, and facilitate access to preferred physician profiles may enhance early presentation and reduce complications.

Normalizing discussion around anorectal symptoms is essential to overcoming embarrassment-related barriers to medical consultation. Public health campaigns such as the TaCa Healthcare “#Let’sTalkPiles” have aimed to reduce stigma through open, relatable messaging. Integration of anorectal health education into broader wellness initiatives or colorectal cancer screening programs may also encourage earlier discussions. Clinicians can contribute by using neutral, non-stigmatizing language during routine consultations (e.g., asking about bowel habits without euphemisms) and by proactively addressing common symptoms without waiting for the patient to raise them. Additionally, social media platforms and online health communities—particularly those led by patients, advocacy groups, or gastroenterology societies—can play a role in demystifying these conditions and promoting health-seeking behavior.

While hemorrhoids are a frequent cause of anorectal symptoms, clinicians should remain vigilant for potentially more severe gastrointestinal pathologies, especially when bleeding is present, to avoid misattributing these signs exclusively to hemorrhoidal disease [15,16]. Diagnostic accuracy in evaluating anorectal conditions remains a significant challenge among healthcare professionals. In a prospective study assessing clinicians’ ability to identify benign anorectal pathology based on clinical images, the overall diagnostic accuracy among physicians was found to be only 53.5% [15,17]. Notably, surgeons demonstrated the highest level of accuracy at 70.4%, while non-surgical specialists scored below 50%, underscoring a potential gap in training and experience regarding anorectal disorders among general practitioners and other specialists. These findings highlight the need for improved medical education and clinical exposure in this domain to ensure timely and appropriate management of patients presenting with anorectal symptoms [15,17,18].

This study has several limitations that should be acknowledged. First, the cross-sectional design precludes any causal inference between the investigated variables and medical consultation behavior. While associations were observed, they cannot be interpreted as predictive or explanatory of longitudinal outcomes.

Second, the data were collected using an online self-administered questionnaire, which introduces potential selection bias. Participants with Internet access and a certain level of digital literacy were more likely to respond, potentially underrepresenting older adults or those from lower socioeconomic backgrounds.

Third, all responses were self-reported, and no clinical confirmation of hemorrhoidal disease was performed. This raises the possibility of misclassification bias, particularly for participants who may have misinterpreted their symptoms or did not have a formal diagnosis.

Fourth, some variables—such as “discomfort discussing anal symptoms” or “preference for physician gender”—are subject to social desirability bias, especially in a culturally sensitive context like Romania. Respondents may have minimized or exaggerated certain responses to align with perceived norms.

Another limitation of this study is the lack of data on how respondents acquired their knowledge about hemorrhoidal disease. In today’s digitally connected society, understanding the sources of health information (e.g., social media, medical websites, and healthcare professionals) would be valuable for designing targeted educational interventions. This represents a key direction for future research.

Finally, although the sample size was adequate for most statistical analyses, certain subgroup comparisons (e.g., by educational level or sex preference) had small cell sizes, which may limit statistical power and generalizability. Also, a larger sample size would have increased the generalizability of the findings.

Future studies using a mixed-methods design, including qualitative interviews and clinical verification, would provide more nuanced insights into the barriers to medical consultation for anorectal symptoms.

## 5. Conclusions

This study provides valuable insight into the behavioral, perceptual, and sociodemographic factors that influence medical consultation for hemorrhoidal symptoms in the general population. Our findings reveal that symptom frequency, perceived severity, and emotional impact significantly affect the likelihood of seeking medical care, while barriers such as embarrassment, fear of diagnosis, or lack of information remain prevalent. Additionally, age and sex, as well as communication comfort and doctor gender preference, appear to play nuanced roles in shaping health-seeking behavior.

These results highlight the ongoing need for targeted health education that sheds light on anorectal conditions, reduces stigma, and encourages timely consultation.

Future interventions should aim to improve patient–physician communication and increase awareness of hemorrhoids as a treatable medical condition, thereby reducing delays in diagnosis and management.

## Figures and Tables

**Table 1:** Characteristics of study participants.

Age (mean ± range)	41.5	(19–91)
Sex—Female	127	68.6%
Sex—Male	58	31.4%
Residence—Urban	152	82.2%
Residence—Rural	33	17.8%
Education—Primary School	8	4.3%
Education—High School	19	10.3%
Education—Post-secondary non-university	1	0.5%
Education—University	102	55.1%
Education—Postgraduate	55	29.7%

**Table 2:** Correlation between participants’ self-perceived understanding of hemorrhoids and various beliefs and behaviors.

Variable		How Well Do You Think You Understand What Hemorrhoids Are?
How severe do you consider a diagnosis of hemorrhoids to be?	Spearman’s rho	0.245
	*p*-value	<0.001
Do you believe hemorrhoids are common in the general population?	Spearman’s rho	−0.334
	*p*-value	<0.001
How likely would you be to recommend a close person to see a doctor for hemorrhoidal symptoms?	Spearman’s rho	0.515
	*p*-value	<0.001
Have you ever used over-the-counter treatments for your symptoms?	Spearman’s rho	−0.109
	*p*-value	0.141

**Table 3:** Mann–Whitney U test on perceived severity of hemorrhoid diagnosis.

**Independent Samples *T*-Test**			
	**U**	**df**	***p***
How severe do you consider a diagnosis of hemorrhoids to be?	4345.500		0.041

*Note:* Mann–Whitney U test.

**Table 4:** Kruskal–Wallis test on perceived understanding of hemorrhoids across education levels.

*Kruskal–Wallis Test*			
Factor	Statistic	Df	*p*
How well do you think you understand what hemorrhoids are?	18.341	4	0.001

**Table 5:** Logistic regression model summary for predicting medical consultation for hemorrhoidal symptoms.

Model	Deviance	AIC	BIC	df	ΔΧ^2^	*p*	McFadden R^2^	Nagelkerke R^2^	Tjur R^2^	Cox & Snell R^2^
M_0_	227.765	229.765	232.980	183	–	–	0.000	0.000	0.000	0.000
M_1_	207.865	223.865	249.585	176	19.900	0.006	0.087	0.144	0.104	0.103

Note: M_0_: intercept-only model; M_1_: full model including age, perceived seriousness of diagnosis, sex, and level of education. Outcome: “Have you consulted a doctor for these symptoms?” (Yes = 1).

**Table 6:** Coefficients for the logistic regression model M_1_.

Predictor	Estimate	Std. Error	z	Wald χ^2^	df	*p*
(Intercept)	−3.548	1.255	−2.827	7.992	1	0.005
Age	0.037	0.014	2.572	6.616	1	0.010
Perceived seriousness of hemorrhoid diagnosis	0.197	0.169	1.171	1.371	1	0.242
Sex (1 = male)	0.900	0.363	2.480	6.151	1	0.013
Education Level (2)	0.129	0.928	0.139	0.019	1	0.889
Education Level (3)	15.432	882.744	0.017	<0.001	1	0.986
Education Level (4)	0.165	0.906	0.182	0.033	1	0.855
Education Level (5)	0.140	0.905	0.155	0.024	1	0.877

**Note:** The reference level for education is category 1. Sex is coded as 1 for male.

**Table 7:** Independent samples test—Frequency of symptoms by medical consultation status.

Independent Samples *T*-Test							
						95% CI for Rank-Biserial Correlation	
	U	df	*p*	Rank-Biserial Correlation	SE Rank-Biserial Correlation	Lower	Upper
How often do these symptoms occur?	2452.500		<0.001	−0.328	0.092	−0.478	−0.158

Note: For the Mann–Whitney test, the effect size is given by the rank biserial correlation. Mann–Whitney U test.

**Table 8:** Mann–Whitney U test for the perceived impact of symptoms on daily life.

Independent Samples *T*-Test							
						95% CI for Rank-Biserial Correlation	
	U	Df	*p*	Rank-Biserial Correlation	SE Rank-Biserial Correlation	Lower	Upper
How bothersome are these symptoms to your daily life?	1973.000		<0.001	−0.459	0.092	−0.590	−0.305

Note: For the Mann–Whitney test, the effect size is given by the rank biserial correlation. Mann–Whitney U test.

**Table 9:** Kruskal–Wallis test on comfort discussing anal symptoms with a doctor.

Kruskal–Wallis Test			
Factor	Statistic	df	*P*
Would you feel comfortable discussing anal symptoms with a doctor?	12.342	2	0.002

## Data Availability

The datasets analyzed during the current study are available from the first and corresponding author upon reasonable request.
